# Psychometric evaluation and reference values for the German Postconcussion Symptom Inventory (PCSI-SR8) in children aged 8–12 years

**DOI:** 10.3389/fneur.2023.166828

**Published:** 2023-11-17

**Authors:** Marina Zeldovich, Leonie Krol, Dagmar Timmermann, Ugne Krenz, Juan Carlos Arango-Lasprilla, Gerard Gioia, Knut Brockmann, Inga K. Koerte, Anna Buchheim, Maike Roediger, Matthias Kieslich, Nicole von Steinbuechel, Katrin Cunitz

**Affiliations:** ^1^Institute of Medical Psychology and Medical Sociology, University Medical Center Goettingen, Goettingen, Germany; ^2^Institute of Psychology, University of Innsbruck, Innsbruck, Austria; ^3^Department of Psychology, Virginia Commonwealth University, Richmond, VA, United States; ^4^Division of Pediatric Neuropsychology, Safe Concussion Outcome Recovery and Education Program, Children's National Health System, Department of Pediatrics and Psychiatry and Behavioral Sciences, George Washington University School of Medicine, Rockville, MA, United States; ^5^Interdisciplinary Pediatric Center for Children with Developmental Disabilities and Severe Chronic Disorders, Department of Pediatrics and Adolescent Medicine, University Medical Center Goettingen, Goettingen, Germany; ^6^Department of Child and Adolescent Psychiatry, Psychosomatics, and Psychotherapy, Ludwig-Maximilians-Universitaet Muenchen, Munich, Germany; ^7^Department of Pediatric and Adolescent Medicine- General Pediatrics- Intensive Care Medicine and Neonatology, University Hospital Muenster, Muenster, Germany; ^8^Department of Paediatric Neurology, Goethe-University Frankfurt/Main, Frankfurt, Germany; ^9^Department of Psychiatry and Psychotherapy, University Medical Center Goettingen, Goettingen, Germany

**Keywords:** pediatric traumatic brain injury, post-concussion symptoms, patient-reported outcome measure (PROM), Postconcussion Symptom Inventory (PCSI), reference values

## Abstract

**Background:**

Post-concussion symptoms (PCS) are a common consequence of pediatric traumatic brain injury (pTBI). They include cognitive, emotional, and physical disturbances. To address the lack of age-adapted instruments assessing PCS after pTBI, this study examines the psychometric properties of the German 17-item post-TBI version of the Postconcussion Symptom Inventory (PCSI-SR8) in children aged 8–12 years. The study also aims to establish reference values based on data from a pediatric general population sample to better estimate the prevalence and clinical relevance of PCS after pTBI in clinical and research settings.

**Methods:**

A total of 132 children aged 8–12 years from a post-acute TBI sample and 1,047 from a general population sample were included in the analyses. The questionnaire was translated from English into German and linguistically validated using forward and backward translation and cognitive debriefing to ensure comprehensibility of the developed version. Reliability and validity were examined; descriptive comparisons were made with the results of the English study. Measurement invariance (MI) analyses between TBI and general population samples were conducted prior to establishing reference values. Factors contributing to the total and scale scores of the PCSI-SR8 were identified using regression analyses. Reference values were calculated using percentiles.

**Results:**

Most children (TBI: 83%; general population: 79%) rated at least one symptom as “a little” bothersome. The German PCSI-SR8 met the psychometric assumptions in both samples and was comparable to the English version. The four-factor structure comprising physical, emotional, cognitive, and fatigue symptoms could be replicated. The MI assumption was retained. Therefore, reference values could be provided to determine the symptom burden of patients in relation to a comparable general population. Clinical relevance of reported symptoms is indicated by a score of 8, which is one standard deviation above the mean of the general population sample.

**Conclusion:**

The German version of the PCSI-SR8 is suitable for assessment of PCS after pTBI. The reference values allow for a more comprehensive evaluation of PCS following pTBI. Future research should focus on validation of the PCSI-SR8 in more acute phases of TBI, psychometric examination of the pre-post version, and child-proxy comparisons.

## 1 Introduction

Pediatric traumatic brain injury (pTBI) is a significant condition with a wide range of incidence worldwide (47–280 per 100,000). The highest rates are reported in Australia and the lowest in Northern Europe (1). In Germany, TBI accounts for 45–80% of all accidental deaths and affects approximately 580 per 100,000 children and adolescents up to the age of 16 (2). While most of the cases are classified as “mild” according to the Glasgow Coma Scale (1, 3) (GCS ≥ 13), approximately one-third of those affected report cognitive, somatic, and/or emotional disturbances, collectively referred to as post-concussion symptoms (PCS) (4). While most symptoms resolve within the first month after the injury, in some cases, PCS may persist for longer (5), affecting the daily lives of patients and their families (6, 7).

To better capture the individual’s perspective on symptom burden, assessment of PCS is often based on self-report (6). Two patient-reported outcome measures (PROMs) listed in the Common Data Elements (CDE) recommendations (8) to measure PCS are the Rivermead Post-Concussion Symptoms Questionnaire (RPQ) (9) for adults and the Postconcussion Symptoms Inventory (PCSI) (10, 11) for children and adolescents.

While the RPQ is available in an age-independent version (16 items), the age-adjusted PCSI consists of three self-report forms and one proxy form. The self-report forms are the PCSI-SR5 (5–7 years; 5 items), the PCSI-SR8 (8–12 years; 17 items), and the PCSI-SR13 (13–18 years; 21 items). The 20-item proxy version (PCSI-P; suitable for ages 5–18) can be used when children and adolescents are unable to complete the questionnaires themselves, to obtain the opinions of caregivers, or to supplement self-reports (11). The items apply a Guttman scale, which differs according to the age version. For children up to 12 years of age, three response categories are used (0: “No”, 1: “A little”, 2: “A lot”). For adolescents and the proxy version, a seven-point scale ranging from 0 to 6 with three anchors (0: “Not a problem”, 3: “Moderate problem”, 6: “Severe problem”) is utilized. All versions refer to two occasions: before (pre version) and after (post version) TBI. The interpretation is based on the Retrospective Adjusted Post-Injury Difference (RAPID) score, which determines clinically significant changes before and after injury. Improvement or worsening of symptom burden is defined as an 80% change that is deemed clinically relevant. In younger children, it is often difficult to obtain reliable self-reported information about pre-TBI symptom experience, especially if the TBI occurred in early childhood. In this case, only the post version can be used (11).

To date, only one instrument for assessing PCS after pTBI has been validated in the German-speaking context, the RPQ in adolescents (13–17 years of age) (12). Validation of the proxy version for younger children (i.e., aged 8–12 years) is currently ongoing. Thus, there is still a need for an age-appropriate German version of the PCSI, since the RPQ was primarily designed to assess PCS in adults (9).

Interpretation of values obtained from PROMs can be challenging. Often, there are no suitable normative or reference data from a comparable general population that can be used to determine the clinical relevance of self-reported symptoms. For the post-TBI version of the PCSI, no such reference data are available either in English- or German-speaking countries. Given the relatively high prevalence of symptoms comparable to PCS in the general pediatric population (13), establishing reference data would facilitate understanding of the clinical relevance and intensity of reported PCS, thus helping clinicians to focus on potentially affected areas.

To fill these gaps, the present study aims to investigate the psychometric properties of the newly translated German post-TBI version of the PCSI-SR8 and to provide reference values for the interpretation of the scores in clinical practice and research.

## 2 Materials and methods

### 2.1 Participants – TBI sample

**Figure 1:**
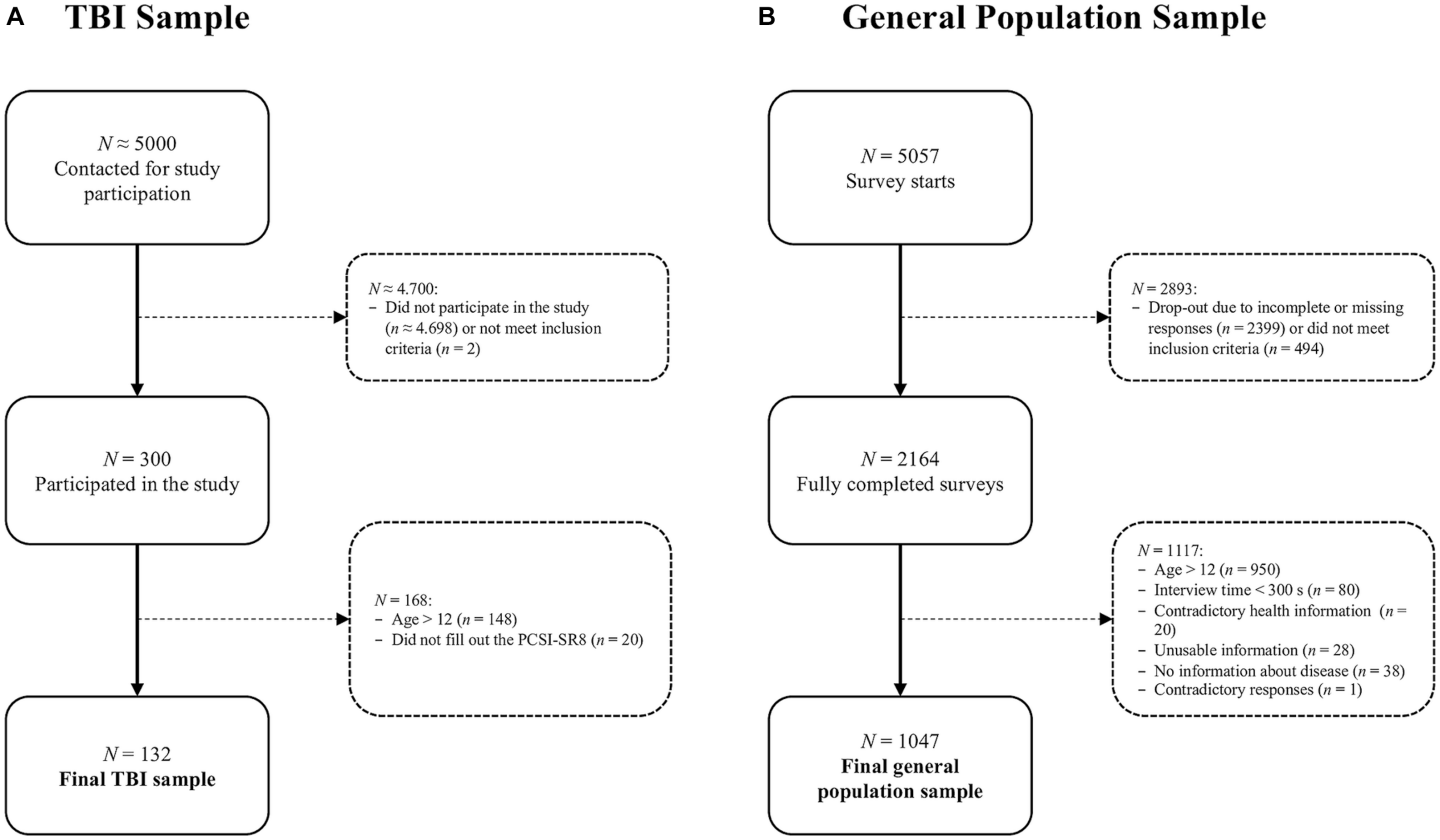
Sample composition: **(A)** TBI sample, **(B)** general population sample.

Participants were recruited from hospital registers in Germany between January 2019 and January 2022. Inclusion criteria for the core study were age 8–17 years, diagnosis of TBI at least three months but no more than ten years prior to study enrolment, formal GCS score or clinically diagnosed TBI severity, outpatient status or being at the beginning of hospital discharge, and ability to understand and respond to the questions. Exclusion criteria were current vegetative state (i.e., minimally conscious according to the GCS), spinal cord injury, severe premorbid mental illness (e.g., psychosis, autism, etc.), epilepsy, terminal illness, or very severe polytrauma. Those who met the inclusion criteria were invited by postal mail to participate in the study (approx. 5,000 invitations). Children, adolescents, and their families were informed of the purpose and procedures of the study. Participants and/or their parents or legal guardians provided written informed consent and medical record release forms. The interviews were conducted in person, either online or offline. From a total of 300 participants aged 8–17 years, 152 children aged 8–12 years were recruited. Overall, 132 children completed the PCSI-SR8. For more details on sample composition, see Figure 1A – TBI sample.

### 2.2 Participants – general population sample

Participants were recruited and surveyed online from March 2022 to April 2022 using the databases of two Germany-based market research agencies.^1^ In the first phase, agencies used information in the database to identify and contact parents of children ages 8 to 17. Recruitment was conducted through email invitations, phone notifications, banners, and messages on panel community pages to engage people with a variety of motivations to participate in the study. They were informed of the purpose of the data assessment and the privacy policy and were asked to explicitly consent to the assessment of sensitive information (i.e., their children’s health data). After providing sociodemographic information, parents were asked whether their child had experienced a TBI or had a serious life-threatening condition. If so, participation in the study was terminated. All other parents were redirected to the socio-demographic questions and then asked if the child was available at the present time. If not, the questionnaire could be completed later; if yes, the child was invited and after a question about readiness to begin, which had to be confirmed, the pediatric questionnaires were presented. Participants received incentives in the form of tokens or certificates.

To ensure data quality, participants who provided inconsistent or unusable information (e.g., children attending vocational school, which is not possible according to the German school system), those with inconsistent responses (e.g., no health problems but a comment in the text box), and those who completed the survey in less than five minutes (i.e., the time required to click through the survey without paying attention to the questions) were excluded. A total 1,047 children aged 8–12 years were included in the analyses from 2,164 completed surveys of children and adolescents. For more details on sample composition, see Figure 1B – General population sample.

#### 2.2.1 Ethical approval

Both studies have been conducted in accordance with all relevant German laws including but not limited to the ICH Harmonized Tripartite Guideline for Good Clinical Practice (“ICH GCP”) and the World Medical Association’s Declaration of Helsinki (“Ethical Principles for Medical Research Involving Human Subjects”). The Ethics Committee of the University Medical Center in Goettingen has approved the studies (application number 19/4/18).

### 2.3 Materials and measures

Sociodemographic and health-related information was collected for both the TBI and general population samples. Participants of the TBI sample or their proxies also completed additional questionnaires measuring health-related quality of life (HRQoL), PCS, anxiety, and depression, which were used in psychometric analyses (see the description of the questionnaires for more details).

#### 2.3.1 Sociodemographic and health-related information

Sociodemographic data comprised age, gender, and school participation. In the TBI sample, health-related information comprised information on health status reported by parents (i.e., presence of chronic health complaints, neurological disorders, and/or developmental health conditions), TBI severity (mild, moderate, severe), time since TBI, and functional recovery/disability status as measured by the King’s Outcome Scale for Childhood Head Injury (KOSCHI) (14). The KOSCHI score includes the following categories: (1) dead, (2) vegetative state, (3a) lower severe disability, (3b) upper severe disability, (4a) lower moderate disability, (4b) upper moderate disability, (5a) good recovery, and (5b) intact recovery.

In the general population sample, information on health status was based on the parental report. It consisted of nine categories, with multiple answers allowed: central nervous system disease; alcohol and/or psychotropic substance abuse; active or uncontrolled systemic disease; psychiatric disorders; severe sensory deficits; use of psychotropic drugs or other medications; intellectual disability or other neurobehavioral disorders; pre, peri-, and postnatal problems; other. If at least one was endorsed, a participant was considered to have at least one chronic health condition.

#### 2.3.2 Postconcussion Symptom Inventory – self report (PCSI-SR8)

The PCSI-SR8 has been translated into German largely in accordance with translation and linguistic validation guidelines (15). Two staff members of the Institute of Medical Psychology and Medical Sociology at the University Medical Center Goettingen independently translated the English version of the PCSI-SR8 into German. With support of a language coordinator (NvS), the two German translations were harmonized. A backward translation into English was then performed by an independent professional translator. The next step was to compare the backward translation with the original English version and, if possible, bring the item wording closer to English. One of the lead developers of the original version of the PCSI (GG) was consulted about any necessary deviations in the wording of individual items resulting from the translation process. The final German version of the PCSI-SR8 was subjected to a cognitive debriefing (CD) with a total of three children aged 8–9 years (one female child after TBI, one female and one male child without TBI). The CD was used to check the comprehensibility of the instructions and the individual items. After evaluating and discussing the CD, no further adjustments were made. In the present study, only PCSI-SR8 post-TBI version was used.

For the general population, the questionnaire was adapted by removing the reference to the TBI. The only change was to the instruction, which was rephrased as follows: “We would like to know if you have any of these complaints at the present time (yesterday and today).” Since there was no further explicit reference to injury, the wording of the items and response categories remained the same.

#### 2.3.3 Rivermead Post-Concussion Symptoms Questionnaire (RPQ) – proxy report

The RPQ (9) is a PROM that assesses 16 PCS rated on a five-point Likert-type scale (from 0: “No problem at all” to 4 “A severe problem”). Although the original scoring included only the total score, there is evidence that the RPQ provides multidimensional assessment of the PCS in adults (16) and adolescents (12). Therefore, in addition to the total score, three scale-scores were used, including somatic (9 items), cognitive (3 items), and emotional (4 items) symptoms. For the calculation of the total and scale scores, values of 1, indicating no more problems with symptoms than before TBI, were treated as 0. Higher score values indicate higher symptom burden. Because the wording of the RPQ items is not age-appropriate for the present study age group, proxies were asked to rate the severity of the children’s symptoms.

#### 2.3.4 Quality of life after brain injury for kids and adolescents (QOLIBRI-Kid/ADO) – self report

The QOLIBRI-KID/ADO (17) is a PROM assessing TBI-related HRQoL in children and adolescents. The final version of the questionnaire comprises 35 items with a five-point response format (from 1: “Not at all” to 5: “Very”) forming six scales (Cognition, Self, Daily Life & Autonomy, Social Relationships, Emotions, and Physical Problems). The total score depicting average values of the scales is transformed in a 0–100 scale with higher values associated with better HRQoL.

#### 2.3.5 Generalized anxiety disorder 7 (GAD-7) – proxy report

The GAD-7 (18) assesses anxiety symptoms using seven items applying a five-point response scale (from 0: “Not at all” to 3: “Nearly every day”). The total score is calculated as a sum of the items and ranges from 0 (no anxiety) to 21 (anxiety symptoms experienced nearly every day). For the present study, proxy-reported GAD-7 was used since the questionnaire had not been yet validated for administration in children aged 8–12 years.

#### 2.3.6 Patient health questionnaire 9 (PHQ-9) – proxy report

The PHQ-9 (19) measures symptoms of major depression with nine items using a five-point response scale (from 0: “Not at all” to 3: “Nearly every day”). The total score ranges from 0 (no depression) to 27 (depressive symptoms reported nearly every day). For the same reasons as for the GAD-7, the PHQ-9 was also completed by proxies.

### 2.4 Statistical analyses

#### 2.4.1 Missing values

To preserve information, missing values in the questionnaires at the scale or total score level were replaced with the scale or total score means, respectively, if more than two-thirds of the responses were available. This resulted in the substitution of five scores for the PCSI-SR8, seven for the RPQ, twelve for the QOLIBRI-KID/ADO, and one value for the PHQ-9.

#### 2.4.2 Psychometric properties of the PCSI-SR8 and its general-population-adapted version

For the TBI and the general population versions of the PCSI-SR8, we first examined items by providing endorsement rates (*n*, %) per response category (additionally stratified by TBI severity for the TBI sample only). The proportions of items rated as at least “a little” bothersome were reported for both samples and compared to those reported in the original English validation study (11). Then, internal consistency was assessed using Cronbach’s alpha on the scale level, supplemented by the McDonald’s ω (0.70–0.95 considered good–excellent, respectively) (20). Corrected-item-total correlations (CITC) were calculated to examine associations between the items with the respective scales. Values greater or equal to 0.30 corresponding to a medium effect (21) were considered acceptable.

For the TBI sample only, convergent and divergent validity were examined using Spearman correlation coefficients. For convergent validity, we used the proxy version of the RPQ, expecting correlations r_S_ ≥ 0.50 corresponding to a large effect size (21) at the total and scale scores level. For divergent validity, we used total scores of the QOLIBRI-KID/ADO, GAD-7, and PHQ-9, with values |0.30| ≤ r_S_ < |0.50| considered acceptable. At the same time, correlation coefficients provide information about the direction of the associations allowing for testing of construct validity. We expected positive relationships with the questionnaires measuring symptom burden (i.e., RPQ, PHQ-9, and GAD-7) and negative associations with the QOLIBRI-KID/ADO, indicating that higher PCS severity is associated with reduced HRQoL.

Finally, we examined the factorial structure in both samples using confirmatory factor analysis (CFA) with robust weighted least squares estimators (WLSMV) for ordinal items (22). We estimated a four-factor model consisting of physical (8 items), emotional (3 items), cognitive (4 items), and fatigue (2 items) symptoms. The goodness of the model fit was evaluated simultaneously using multiple fit indices, with the desired values given in parentheses: the $\chi^{2}$ -test value (*p* > 0.05), ratio of $\chi^{2}$ value and degrees of freedom ( $\chi^{2}$ /df ≤ 2) (23), comparative fit index (CFI ≥ 0.95) (24), Tucker-Lewis index (TLI ≥ 0.95) (24), root mean square error of approximation (excellent fit RMSEA <0.05; mediocre fit 0.05 ≤ RMSEA <0.10) (25, 26) including 90% confidence interval (CI_90%_), and standardized root mean square residual (SRMR <0.08) (24). To provide robust results, scaled values were obtain for all fit indices except the SRMR (not computable). Where available, the results from the original English PCSI-SR8 study (11) have been provided to allow for a direct comparison.

#### 2.4.3 Reference values for the PCSI-SR8

##### 2.4.3.1 Measurement invariance analyses

Ensuring that the same latent construct of PCS is measured in the general population sample as in the TBI sample is necessary to provide reference values. Therefore, we conducted measurement invariance (MI) testing. We used a CFA framework and estimated three models with imposing parameter constraints following approach suitable for ordinal data (27, 28). First, the baseline model was estimated to account for (1) configural MI. Then, equality of (2) thresholds between the groups was assumed. Finally, equality of (3) thresholds and loadings between the samples was tested. The models were compared using scaled $\chi^{2}$ difference test, with a non-significant result (*p* > 0.05) suggesting no difference between the models tested and thus no violation of the MI assumption. In addition, differences in fit indices were considered with ΔCFI <0.01 (29) and ΔRMSEA ≤0.01 (30) indicating model equivalence. Maintaining this assumption would imply that differences between TBI and general population samples are due solely to differences in the experience of PCS and that meaningful comparisons can be made.

##### 2.4.3.2 Regression analyses

Regression analyses were used to examine the potential effects of other factors for possible further stratification of the reference values. Negative binomial models were estimated to account for the distribution of the PCSI-SR8 scores. We used the PCSI-SR8 total and scale scores as dependent variables and sex, age, and health status as covariates. Second order interactions between covariates were also included in the model.

##### 2.4.3.3 Reference values

Reference values were calculated using percentiles. Percentiles represent the value below which a certain percentage of observations fall. This information helps to determine whether a post-TBI child’s PCSI-SR8 score is below, at, or above the reference population score. The following percentiles were provided: 2.5, 5, 16, 30, 40, 50, 60, 70, 85, 95, and 97.5%. Values that exceed the reference mean (i.e., 50%) by one standard deviation, corresponding to the 85th percentile (rounded up to the next integer) for normally distributed data, were considered clinically relevant. Examples of interpretation are provided in the results section.

##### 2.4.3.4 Software

All analyses were carried out with R version 4.2.3 (31) under application of the packages table1 (32) for descriptive statistics, psych (33) for psychometric analyses, and lavaan (34) for the CFA and the MI analyses. The α-level was set at 5%. Bonferroni correction (i.e., α_adj_ = 0.05/4 = 0.0125) was applied for multiple scale comparisons where appropriate.

## 3 Results

### 3.1 Sample characteristics

The TBI sample included *N* = 132 children (59.8% male) with a mean age of 10.7 ± 1.40 years. The majority reported no chronic health conditions (59.8%). Most suffered a TBI due to a fall (68.2%) without loss of consciousness (74.2%) with the injury having occurred on average 4.49 ± 2.63 years ago. In most cases, the TBI was classified as mild (72.0%) with no lesions present (74%). At the time of study entry, most participants had fully recovered from the injury (88.6%) according to the KOSCHI score. A descriptive comparison to the study investigating psychometric properties of the PCSI-SR8 (11) revealed some differences in the sample composition. The participants of the original English study rather sustained a sport related TBI (41.0%) classified as mild (100%) and were at an early stage of the injury with the mean of 11.3 ± 2.63 days. The general population sample consisted of *N* = 1,047 children (50.0% male) with a mean age of 10.0 ± 1.42 years. Most of the children had no chronic health conditions (88.3%). For more details, see Table 1.

**Table 1:** Sample characteristics of the TBI and general population samples in the present study and comparison with the sample used in the original English validation study.

Variable	Group/value	TBI sample (*N* = 132)	General population sample (*N* = 1,047)	Original English study^1^ (*N* = 315)
Sex	Male	79 (59.8%)	524 (50.0%)	n.a.
	Female	53 (40.2%)	523 (50.0%)	n.a.
Age (years)	*M* (*SD*)	10.7 (1.41)	10.0 (1.42)	n.a.
	*Md* [*Min*, *Max*]	10.5 [8.00, 12.9]	10.0 [8.00, 12.0]	n.a.
Education	None	13 (9.8%)	0 (0%)	n.a.
	Nursery	36 (27.3%)	n.a.	n.a.
	Primary school	55 (41.7%)	556 (53.1%)	n.a.
	Special school	1 (0.8%)	47 (4.5%)	n.a.
	Secondary school	11 (8.3%)	236 (22.5%)	n.a.
	Preparatory high school	2 (1.5%)	193 (18.4%)	n.a.
	Missing	14 (10.6%)	15 (1.4%)	n.a.
Integration assistance	Yes	1 (0.8%)	145 (13.8%)	n.a.
	No	127 (96.2%)	902 (86.2%)	n.a.
	Missing	4 (3.0%)	0 (0%)	n.a.
Chronic health conditions^2^	One and more	79 (59.8%)	122 (11.7%)	-
	None	53 (40.2%)	925 (88.3%)	-
Injury cause	Sports	n.a.	-	130 (41%)
	Fall	90 (68.2%)	-	83 (26%)
	Road traffic/Motor vehicle	17 (12.9%)	-	40 (13%)
	Crash with an object	3 (2.3%)	-	-
	Other	22 (16.6%)	-	58 (18%)
	Missing/not reported	0 (0%)	-	4 (1%)
Loss of consciousness	No	98 (74.2%)	-	192 (61%)
	Yes	33 (25.0%)	-	89 (28%)
	Missing	1 (0.8%)	-	34 (11%)
Time since injury	*M* (*SD*)	4.49 (2.63)^3^	-	11.3 (7.4)^4^
TBI severity	Mild	95 (72.0%)	-	315 (100%)
	Moderate	11 (8.3%)	-	-
	Severe	26 (19.7%)	-	-
Lesions	No	97 (73.5%)	-	n.a.
	Yes	34 (25.8%)	-	n.a.
	Missing	1 (0.8%)	-	n.a.
Recovery (KOSCHI)	4a	3 (2.3%)	-	n.a.
	4b	2 (1.5%)	-	n.a.
	5a	10 (7.6%)	-	n.a.
	5b	117 (88.6%)	-	n.a.

^1^Values obtained from the original validation study by Sady et al. (11). Sex was reported without age stratification and thus cannot be provided. Age was reported in groups only (i.e., 8–12 years). ^2^Presence of chronic health conditions is based on parental report and includes at least one health problem from the following lists: presence of chronic health complaints, neurologic disorders, and/or developmental health conditions (TBI sample) and central nervous system disease, alcohol and/or psychotropic substance abuse, active or uncontrolled systemic disease, psychiatric disorders, severe sensory deficits, use of psychotropic drugs or other medications, intellectual disability or other neurobehavioral disorders, pre, peri-, and postnatal problems, other (general population sample). ^3^Time since injury in years. ^4^Time since injury in days. n.a., information is not available or not collected in this form; −, information is not relevant for this sample; *N*, absolute frequencies %:, relative frequencies; *M*, mean; *SD*, standard deviation; *Md*, median; *Min*, minimum; *Max*, maximum; TBI, traumatic brain injury; KOSCHI, King’s Outcome Scale for Closed Head Injury (4a: lower moderate disability; 4b: upper moderate disability; 5a: good recovery; 5b: intact recovery). Values may not add up to 100% due to rounding.

### 3.2 Response patterns

Overall, most children (TBI: 83%; general population: 79%) rated at least one symptom as “a little” bothersome, including 24 and 17%, respectively, who rated at least one symptom as “very” bothersome. Analysis of item response patterns revealed that participants in both the TBI and general population samples were less likely to report the maximum level of PCS (i.e., the “a lot” category was selected less frequently for all items). Frequencies in this category ranged from 3% (*Difficulty remembering*) to 6% (*Headaches*) in the TBI sample and from 0.4% (*Feeling slowed down*) to 2.8% (*Irritability*) in the general population sample. When TBI severity groups were examined separately, no systematic differences in the distribution of responses due to group size were observed, at least on a descriptive basis. However, participants in the mild TBI subsample were more likely to endorse emotional and fatigue items, whereas participants in the severe TBI subsample tended to rate physical and cognitive symptoms more highly. Comparison with the response patterns of the original English study sample showed a higher frequency of “a lot” responses, particularly for symptoms such as *Headache* (28%) and *Fatigue* (23%), and less frequently reported symptoms such as *Blurred vision* (2%) or *Nervousness* (6%). For a detailed overview, see Supplementary Table S1.

**Figure 2:**
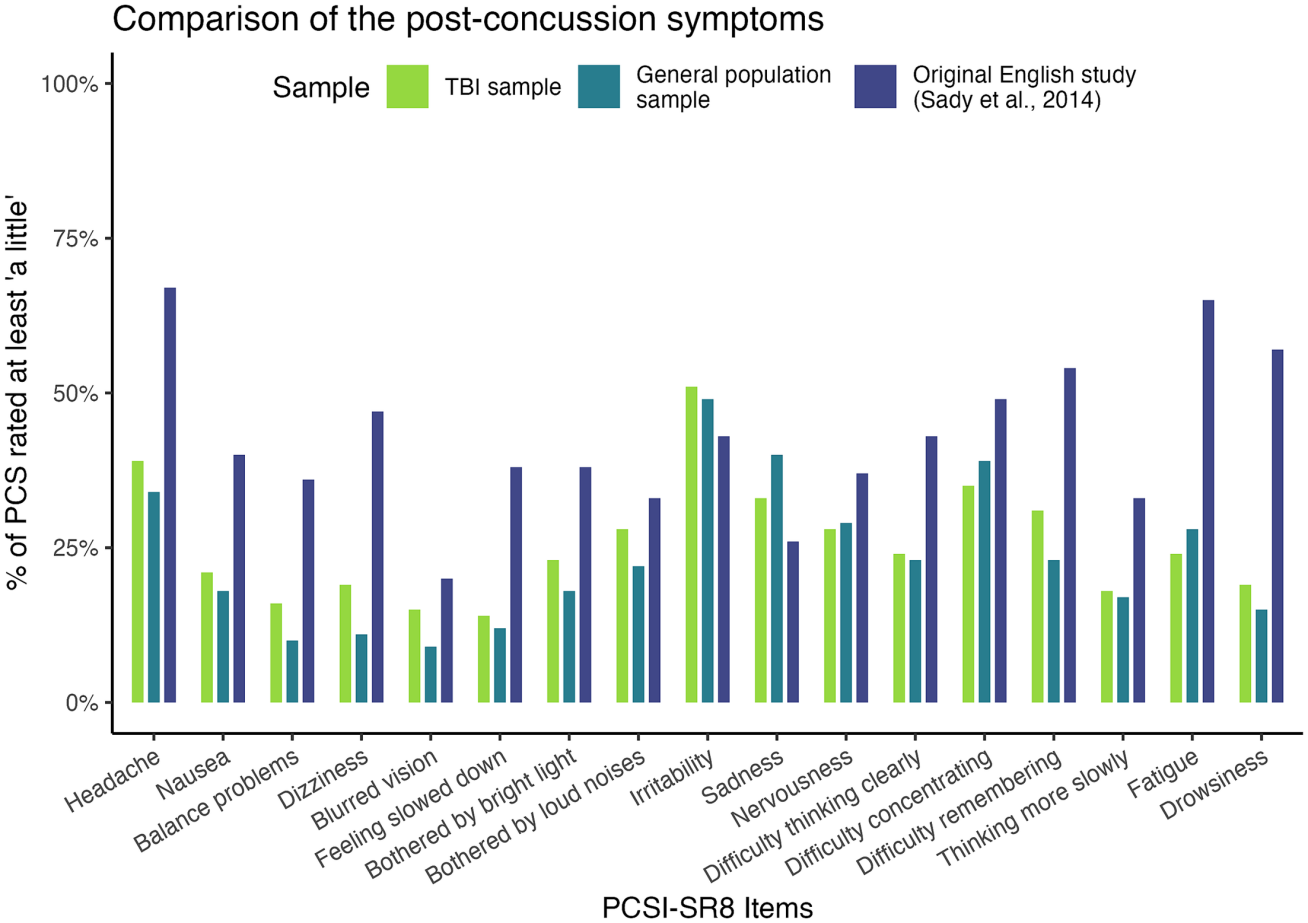
Proportions of PCS rated as at least “a little”.

Figure 2 provides an overview of symptoms rated as at least “a little.” We found a trend toward more bothersome PCS in the acute mild TBI sample used to validate the English version of the PCSI-SR8, followed by the post-acute TBI sample used in the present study and the general population sample in all scales except the emotional symptom scale. For emotional symptoms (particularly irritability and sadness), both our TBI sample and the general population sample were more likely to be bothered.

### 3.3 Reliability

Table 2 provides reliability coefficients for the PCSI-SR8 in the TBI and general population samples and for the original English study. Cronbach’s ɑ and McDonald’s ɷ ranged from ɑ = 0.69 and ɷ = 0.73 (Emotional scale) to ɑ = 0.90 and ɷ = 0.91 (Total score) in the TBI sample and from ɑ = 0.66 and ɷ = 0.68 (Emotional scale) to ɑ = 0.89 and ɷ = 0.90 (Total score) in the general population sample. The Cronbach’s ɑ values were comparable to those reported in the original study. The omission of none of the items resulted in exceeding the initial Cronbach’s ɑ of the respective scale. The CITCs were above 0.40 in both samples.

**Table 2:** Results of reliability analyses.

		TBI sample				General population sample				Original English version^1^
Scale	Item	Cronbach’s ɑ	McDonald’s ɷ	Cronbach’s ɑ if item omitted	CITC	Cronbach’s ɑ	McDonald’s ɷ	Cronbach’s ɑ if item omitted	CITC	Cronbach’s ɑ
Physical	Headache	**0.79**	**0.83**	**0.78**	**0.45**	**0.85**	**0.86**	**0.84**	**0.51**	**0.81**
	Nausea			**0.75**	**0.65**			**0.83**	**0.58**	
	Balance problems			**0.76**	**0.61**			**0.82**	**0.66**	
	Dizziness			**0.76**	**0.62**			**0.82**	**0.65**	
	Blurred vision			**0.75**	**0.65**			**0.83**	**0.57**	
	Feeling slowed down			**0.76**	**0.56**			**0.83**	**0.57**	
	Sensitivity to light			**0.79**	**0.42**			**0.83**	**0.58**	
	Sensitivity to noise			**0.77**	**0.55**			**0.84**	**0.52**	
Emotional	Irritability	0.69	**0.73**	**0.66**	**0.55**	0.66	0.68	**0.64**	**0.42**	0.62
	Sadness			**0.66**	**0.56**			**0.48**	**0.53**	
	Nervousness			**0.44**	**0.74**			**0.56**	**0.47**	
Cognitive	Difficulty thinking clearly	**0.73**	**0.80**	**0.65**	**0.66**	**0.79**	**0.84**	**0.70**	**0.66**	**0.84**
	Difficulty concentrating			**0.70**	**0.57**			**0.74**	**0.57**	
	Difficulty remembering			**0.69**	**0.57**			**0.77**	**0.52**	
	Thinking more slowly			**0.65**	**0.67**			**0.72**	**0.61**	
Fatigue	Fatigue	**0.74**	**0.74**	**-**	**-**	**0.76**	**0.76**	-	-	**0.79**
	Drowsiness			**-**	**-**			-	-	
Total		**0.90**	**0.91**	**-**	**-**	**0.89**	**0.90**	-	-	**0.90**

^1^Values obtained from the original validation study by Sady et al. (11). CITC, corrected item-total correlations. The values printed in **bold** are within the permissible cut-off values (Cronbach’s ɑ and McDonald’s ɷ: ≥ 0.70, Cronbach’s ɑ if item omitted does not exceed Cronbach’s ɑ; CITC: ≥ 0.30). Cronbach’s ɑ if item omitted and CITC are reported for scales with k > 2 items.

### 3.4 Validity

#### 3.4.1 Factorial validity

Goodness of fit indices indicated replicability of the original four-factor structure with *χ*^2^ (113) = 16.31, *p* = 0.397, *χ*^2^/df = 1.03, CFI = 0.99, TLI = 0.99, RMSEA [90% CI] = 0.015 [0.000, 0.047], SRMR = 0.076 (TBI sample) and *χ*^2^ (113) = 272.11, *χ*^2^/df = 2.41, CFI = 0.99, TLI = 0.98, RMSEA [90% CI] = 0.037 [0.031, 0.042], SRMR = 0.042 (see Table 3).

**Table 3:** Factorial validity.

Sample	*χ*^2^ (df)	*p*	χ^2^/*df*	CFI	TLI	RMSEA [90% CI]	SRMR
TBI sample	16.31 (113)	**0.397**	**1.03**	**0.99**	**0.99**	**0.015 [0.000, 0.047]**	**0.076**
General population sample	272.11 (113)	**<0.001**	2.41	**0.99**	**0.98**	**0.037 [0.031, 0.042]**	**0.042**
Original English version^1^	n.a.	n.a.	2.10	**0.97**	n.a.	**0.065** [n.a., n.a.]	n.a.

^1^Values obtained from the original validation study by Sady et al. (11). n.a., information is not available; *χ*^2^, chi-square statistics; *df*, degrees of freedom (cut-off: ≤ 2); *p*, value of *p*; CFI, Comparative Fit Index (cut-off: > 0.95); TLI, Tucker-Lewis Index (cut-off: > 0.95); RMSEA [90%CI], root mean square error of approximation with 90% confidence interval (cut-off: < 0.06); SRMR, Standardized Root Mean Square Residual (cut-off: < 0.08). Values in **bold** are within acceptable range.

#### 3.4.2 Convergent, divergent, and construct validity

**Figure 3:**
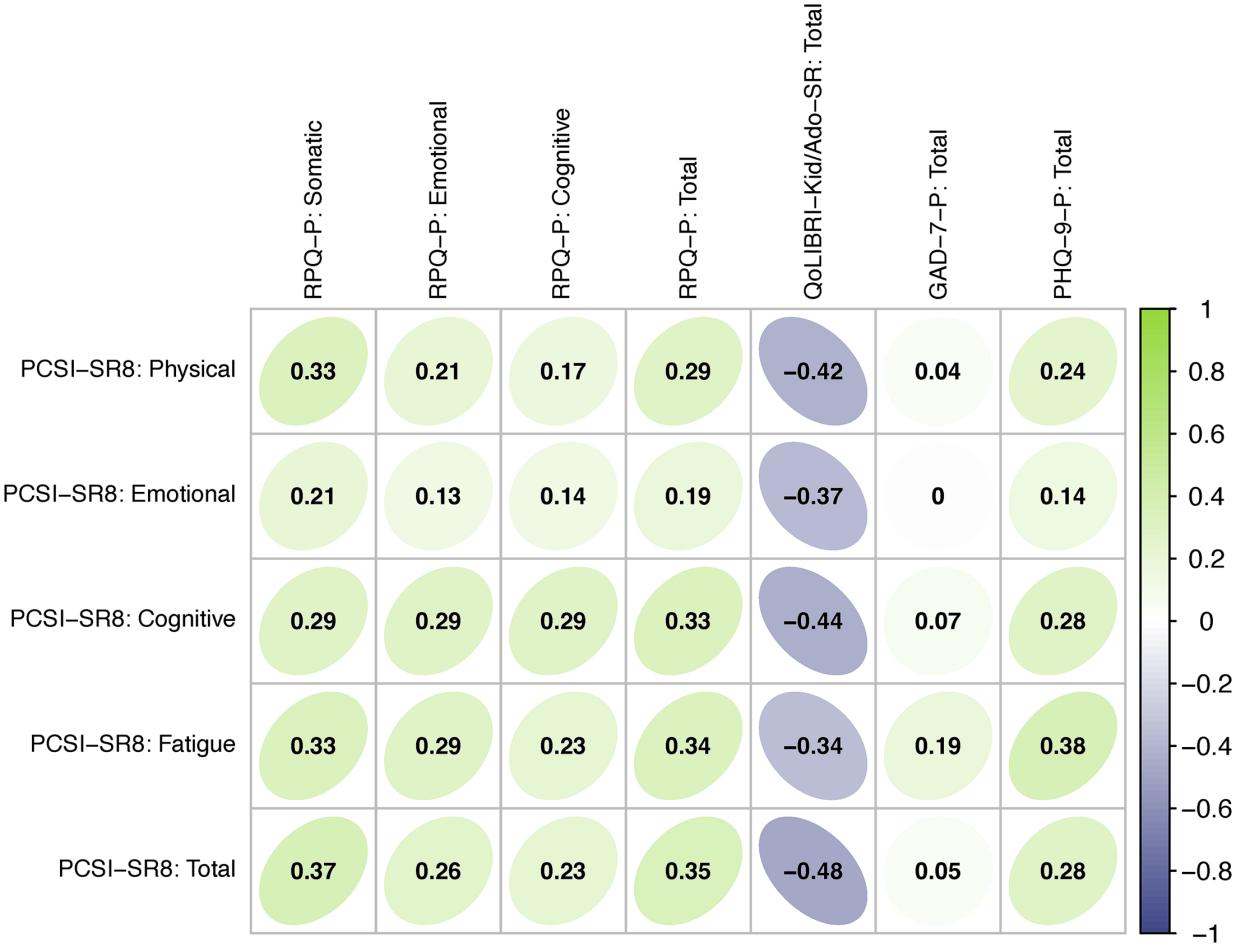
Convergent and divergent validity: Spearman correlations between PROMs. Green shading indicates positive correlations, purple shading indicates negative coefficients.

Figure 3 shows a correlation matrix for the PCSI-SR8, RPQ, QOLIBRI-KID/ADO, GAD-7 and PHQ-9 based on data from the TBI sample. The PCSI-SR8 total and scale scores showed low to moderate positive correlations with the proxy-reported RPQ total and scale scores ranging from 0.13 (Emotional scales) to 0.35 (Total scores). Similarly, low to medium positive associations were observed between the PCSI-SR8 total and scale scores and the proxy-reported PHQ-9 (0.14 for Emotional scale to 0.38 Fatigue scale) and GAD-7 (< 0.01 for Emotional scale to 0.19 Fatigue scale) total scores. Correlations between the PCSI-SR8 total and scale scores and the QOLIBRI-KID/ADO total score were low to medium and negative (−0.34 for Fatigue scale to −0.48 Total score).

### 3.5 Measurement invariance

The MI analyses demonstrated no significant differences between the models with increased constraints, indicating the comparability of the TBI and general population samples in assessing PCS using the PCSI-SR8. For details, see Table 4.

**Table 4:** Measurement invariance analyses.

Samples	Constraints	*χ*^2^ (df)	*p*	*χ*^2^/df	CFI	TLI	RMSEA [90% CI]	SRMR	*χ*^2^ (df)	Δ *χ*^2^	Δ df	*p*
General population vs. TBI sample	Baseline	356.43 (226)	**<0.001**	**1.58**	**0.99**	**0.99**	**0.031 [0.025, 0.037]**	**0.045**	-	-	-	-
	Thresholds	356.43 (226)	**<0.001**	**1.58**	**0.99**	**0.99**	**0.031 [0.025, 0.037]**	**0.045**	234.09 (226)	-	-	-
	Thresholds and loadings	366.35 (239)	**<0.001**	**1.53**	**0.99**	**0.99**	**0.030 [0.024, 0.036]**	**0.045**	247.86 (239)	16.657	13	0.216

*χ*^2^, scaled chi-square statistics; df, scaled degrees of freedom; *p*, value of *p*; *χ*^2^/df, scaled ratio (cut-off: ≤ 2); CFI, scaled Comparative Fit Index (cut-off: > 0.90); TLI, scaled Tucker-Lewis Index (cut-off: > 0.95); RMSEA [90%CI], scaled root mean square error of approximation with 90% confidence interval (cut-off: < 0.06); SRMR, scaled Standardized Root Mean Square Residual (cut-off: < 0.08). Values in bold indicate at least satisfactory/mediocre model fit according to the respective cut-offs and/or are within acceptable range. Only threshold and threshold and loadings models were compared due to identical test values (i.e., *χ*^2^ and *df*), in baseline and threshold models.

### 3.6 Regression analyses

Regression analyses revealed that only health status was significantly associated with PCSI-SR8 total and scale scores (see Table 5). The second-order interaction analyses showed a significant interaction between age and health status only in the Emotional scale. For details, see Supplementary Table S2. These results suggest that reference values should be stratified by health status (i.e., no chronic health complaints vs. at least one chronic health complaint). However, in view of the relatively small number of participants from general population samples with any health conditions (*n* = 122), and thus the potential impact on the generalizability of the results, we decided to exclude this subgroup from the calculation of reference values.

**Table 5:** Results of negative binomial regression analyses.

	Variable	Reference Group	Estimate	S.E.	*z*	*p*
Total score	(Intercept)	-	2.22	0.26	8.44	**<0.001**
	Age	-	−0.02	0.02	−0.80	0.421
	Male	Female	−0.12	0.07	−1.66	0.097
	No chronic health complaints	At least one chronic health complaint	−0.57	0.11	−5.40	**<0.001**
Physical	(Intercept)	-	1.14	0.36	3.14	**0.002**
	Age	-	−0.02	0.03	−0.53	0.596
	Male	Female	−0.13	0.10	−1.33	0.185
	No chronic health complaints	At least one chronic health complaint	−0.58	0.14	−4.04	<0.001*
Emotional	(Intercept)	-	0.91	0.23	3.94	**<0.001**
	Age	-	−0.03	0.02	−1.15	0.250
	Male	Female	−0.13	0.06	−2.15	**0.032**
	No chronic health complaints	At least one chronic health complaint	−0.39	0.09	−4.46	<0.001*
Cognitive	(Intercept)	-	1.06	0.33	3.16	**0.002**
	Age	-	−0.02	0.03	−0.74	0.461
	Male	Female	−0.09	0.09	−1.02	0.306
	No chronic health complaints	At least one chronic health complaint	−0.78	0.13	−6.13	<0.001*
Fatigue	(Intercept)	-	−0.46	0.42	−1.08	0.281
	Age	-	0.02	0.04	0.41	0.681
	Male	Female	−0.07	0.11	−0.61	0.540
	No chronic health complaints	At least one chronic health complaint	−0.48	0.16	−2.98	0.003*

Estimate, regression coefficient; S.E., standard error; *z*, *z*-value; *p*, value of *p*; values in bold are significant at 5%. Covariates additionally marked with an asterisk (*) are significant at 1.25% (adjusted significance level after Bonferroni correction: α_adj_ = 0.05/4 = 0.0125).

### 3.7 Reference values

Table 6 provides reference values for the PCSI-SR8 total and scale scores. The following examples illustrate how this table can be used to interpret a patient’s score after pediatric TBI. Suppose a child after a TBI scores 5 on the PCSI-SR8 total score. Compared to a general population sample, his or her score falls between the 70th and 80th percentiles. The score can be considered average and there is no evidence of clinical relevance of the reported symptom burden. If a child after a TBI scores 15 on the PCSI-SR8 total score, his or her score falls between the 95th and 97.5th percentiles of the general population sample and is therefore above average. The symptom burden can be considered clinically relevant and further diagnosis and treatment is highly indicated. The PCSI-SR8 scale scores can be handled in a similar manner. In this case, a specific symptom domain (e.g., emotional or physical) can be examined for clinical relevance to further refine possible areas of concern.

**Table 6:** Reference values.

		Low symptoms severity			−1 *SD*					*Md*					+1 *SD*			High symptoms severity
		Scale
*N*
2.5%	5%	16%	30%	40%	50%	60%	70%	85%	95%	97.5%
Total	925	0	0	0	1	2	3	3	4	8	14	17
Physical		0	0	0	0	0	0	1	1	3	7	8
Emotional		0	0	0	0	1	1	1	2	3	3	4
Cognitive		0	0	0	0	0	0	1	1	2	4	5
Fatigue		0	0	0	0	0	0	0	0	1	2	3

*N*: absolute frequencies (*n* = 122 participants from the general population sample suffering at least from one chronic health condition are excluded); 50% percentiles represent 50% of the distribution corresponding to the median (*Md*); *SD*, standard deviation (corresponding to the cut-offs of the normal distribution); values from −1 standard deviation (16%) to +1 standard deviation (85%, round up to the next integer) are within the normal range (i.e., not clinically relevant symptom severity); values below 16% indicate low symptoms severity (i.e., absence of PCSI-SR8 symptoms) and values above 85% indicate high symptom severity (i.e., presence of clinically relevant PCSI-SR8 symptoms).

Alternatively, a cut-off of 8 can be used to determine the clinical relevance of the reported symptom burden, which represents the upper limit of one standard deviation above the mean. Scores above this level are considered clinically relevant (i.e., less likely to be reported by the healthy reference population). The same applies for the scale scores. A score of 3 can be used for the Physical and Emotional scales, a score of 2 for the Cognitive scale, and a score of 1 for the Fatigue scale.

## 4 Discussion

The aim of the present study was to investigate the psychometric properties of the age-adapted German version of the PROM for measuring PCS in children aged 8–12 years after TBI (i.e., PCSI-SR8) and to provide reference values for the interpretation of individual patient scores. Our results indicate that the German translation of the PCSI-SR8 is psychometrically comparable to the original English version and can be used for the assessment of PCS after pTBI. The reference values provided allow for an easy interpretation of the PCSI-SR8 scores at the total and scale score level and help to determine the clinical relevance of the PCS exposure. However, some of the results deserve special attention and are discussed in more detail below.

### 4.1 Psychometric properties and comparability with the original English version

The German version of the PCSI-SR8 adapted both positive and negative qualities of the original version. For example, while the reliability coefficients of the Physical, Cognitive, Fatigue, and Total scales indicated good internal consistency, the Emotional scale was the one with values below 0.70 in both TBI and general population samples. This was also the case in the analyses of the original English version reported by Sady et al. (11), where the Emotional scale had the lowest reliability. According to a systematic review of psychometric studies on instruments assessing PCS in student athletes (10), the reliability of instruments used in children aged 5–12 years has not been systematically reported. Therefore, it is challenging to conclude whether the lower internal consistency of the Emotional scale is a maladaptive psychometric characteristic of the PCSI-SR8 or whether this also applies to other questionnaires. A possible explanation could be that children experience challenges in reporting their internalized emotional state, which has been discussed in the literature (35–37).

The results of the correlation analyses indicated the expected direction (i.e., positive associations with scales that measure symptom burden and negative associations with self-reported TBI-specific HRQoL). However, the correlations between some constructs – particularly with an alternative measure, the RPQ, to assess PCS – were rather low. This finding may be explained by the use of proxy reports in assessing depression, anxiety, and PCS using the RPQ. Although proxy reports are commonly used to assess children’s health, they often tend to distort the true condition (38). Specifically, ratings of emotional symptoms show poor parent–child agreement, whereas ratings of physical symptoms do concur (39, 40). Furthermore, parents’ unawareness of certain emotional symptoms or children’s unwillingness or inability to verbalize certain symptoms could affect the quality of the assessment (35, 37, 41). Particularly in the assessment of PCS, which is characterized by a relatively high number of non-directly observable emotional symptoms, child-proxy concordance tends to be moderate (10, 41, 42). A recent study assessing PCS in adolescents aged 13–17 years using both the self-report and proxy-report versions of the RPQ found poor to moderate agreement between ratings (12). The use of the PCSI-P version may have resulted in higher correlations due to greater item concordance between the PCSI-SR8 and the PCSI-P compared to the RPQ. However, the intent was to administer an instrument other than the PCSI, but generally measuring the same construct, to test for convergent validity. The need to treat proxy information with caution, especially in the post-acute phase of injury when awareness of symptom burden may have dissipated over time, and to interview children whenever possible is reinforced by the results of the current study.

### 4.2 Reference values and symptom burden in general population samples

Based on parental report, 11.7% of children in the general population sample had at least one chronic health condition. Health status was the only significant factor contributing to the PCSI-SR8 total and scale scores (i.e., the presence of chronic health conditions was positively associated with symptom burden). This provides an indication of the impact of general health on the development of PC-related symptoms. Given the relatively small sample size of this group, we were not able to stratify the reference values according to the health status. However, this would be a very important point as children with TBI may also have suffered from premorbid conditions. In our study, 40.2% of the children had at least one chronic health condition, as reported by their parents. It is known from adult research (43) that general population samples with at least one chronic health condition are more likely to report PC-like symptoms than those without any chronic conditions. Moreover, some studies in adult context suggest that the development of PCS is TBI independent and is rather associated with the general health state [e.g., (44, 45)]. In the pediatric context, young uninjured athletes aged 9–12 years reported overall increased levels of drowsiness, nervousness, and feeling tired or less able to concentrate (endorsement of the “a little” category ranged from 10 to 27%) (13). However, those with a history of concussion were significantly more affected by cognitive and fatigue symptoms than children without a TBI. In the present study, a history of TBI was one of the exclusion criteria for the general population sample. Therefore, this information could not be included in the reference values. Further investigation of children with chronic health conditions, including concussion history, in general population samples is highly recommended to better determine the clinical relevance of reported PCS after TBI. Finally, given that children between 8 and 12 years of age often experience a prepubertal phase, symptoms such as fatigue (46) or mental health issues (47) may occur. Knowledge of this is therefore crucial for the differential diagnosis and subsequent treatment of PCS.

In summary, the provided reference values should serve as an ideal health norm for the screening of PCS in children after TBI in Germany in order to obtain information for a screening diagnosis. Finally, we recommend that scale scores should always be considered when interpreting symptom burden using the reference values provided. In some cases, a clinical cut-off according to the total score may be in the normal range, but the scale score may exceed levels acceptable for the non-clinical population.

### 4.3 Strengths and limitations

The main strength of the present study is that it is the first study to perform psychometric analyses of an age-adjusted PROM to assess PCS after TBI in children aged 8–12 years and to provide reference values from the general population in Germany. However, there are some limitations to be noted.

First, our sample size of individuals after TBI was relatively small, which may limit the generalizability of the findings. Despite a relatively large pool of families contacted, the response rate was rather low, which might have had several reasons. This limitation has already been exhaustively discussed by von Steinbuechel et al. (17). In addition, our sample consisted of children who had sustained TBI relatively long ago. Therefore, validation of the German PCSI-SR8 at more acute stages after injury, similar to the investigation by Sady et al. (11), is highly indicated. Furthermore, PCS are most common after mild to moderate TBI. Although comparable symptoms are often reported after moderate and severe TBI (9), they are not necessarily due to the brain injury but may have other causes (e.g., extracranial injury or polytrauma). Although our sample consisted predominantly of injuries classified as mild (72%), the absolute number of participants would not be sufficient to perform robust analyses. Therefore, we would strongly recommend further validation of the PCSI-SR8 within different TBI severity groups. Finally, the translated and original versions were only compared descriptively using goodness-of-fit indices according to the respective cut-offs. For further validation of the PCSI-SR8, direct comparisons between language versions (e.g., using MI analyses) would be beneficial to provide evidence of the comparability of the PCS assessment. This would allow data aggregation between language samples and the conduct of multi-center, multi-lingual studies. The same is also applicable to TBI severity groups: MI analyses would provide further evidence that the construct of PCS captured by the PCSI-SR8 is measured equally across the full spectrum of TBI severity, suggesting that differences in questionnaire scores correspond to true differences in experienced symptom burden.

Second, as already stated above, we used proxy measures of mental health (i.e., PHQ-9 and GAD-7) and PCS (i.e., the RPQ) rather than self-report measures, which may have influenced the results of divergent and convergent validity. Because proxy ratings may not fully capture symptom burden, especially when measuring mental and emotional state, further validation of the PCSI-SR8 using self-report information is warranted. Finally, we collected data from the general pediatric population through an online survey, which may have limited the generalizability of our findings to individuals who have access to the Internet and are willing to participate in online surveys. In addition, the exclusion of children with chronic health conditions may have limited the generalizability of our findings to the broader population of children after TBI, and further research is needed to better understand the impact of chronic health conditions on PCS.

### 4.4 Outlook

Further studies to validate the German version of the PCSI should focus on more acute TBI samples, concordance with PCSI-P results, and validation of the pre-post version. For the latter, it would be also beneficial to collect data from TBI populations who have recently sustained a TBI to avoid recall and memory bias. In addition, detailed comparisons between the PCSI-SR8 and the RPQ would provide more insight into the potential benefits of age-adjusted PCS assessments after TBI.

## Data availability statement

The data presented in this study are available upon request from the corresponding author. Data are not publicly available for privacy reasons. R scripts are available from GitHub https://github.com/mzeldovich/Project-Reference-values (last access on 23.07.23). The R-Shiny application with reference values is available at https://reference-values.shinyapps.io/Tables_Reference_values/ (tab PCSI-SR8; last access on 23.07.23).

## Ethics statement

Both studies involving humans were approved by Ethics Committee of the University Medical Center in Goettingen (application number 19/4/18). The studies were conducted in accordance with the local legislation and institutional requirements. Written informed consent for participation in this study was provided by the participants’ legal guardians/next of kin.

## Author contributions

MZ: Conceptualization, Funding acquisition, Fromal analysis, Software, Writing - original draft, Writing - review & editing. LK: Data curation, Formal analysis, Software, Writing – original draft. DT: Investigation, Writing – review & editing. UK: Writing – review & editing. JA-L: Writing – review & editing. GG: Writing – review & editing. KB: Writing – review & editing. IK: Investigation, Writing – review & editing. AB: Writing – review & editing. MR: Investigation, Writing – review & editing. MK: Investigation, Writing – review & editing. NS: Conceptualization, Funding acquisition, Investigation, Writing – review & editing. KC: Data curation, Investigation, Project administration, Writing – original draft, Writing – review & editing.

## Footnotes

^1^Dynata: https://www.dynata.com; respondi: https://www.respondi.com; last access 23.07.2023.
