# Corrigendum: Psychometric evaluation and reference values for the German Postconcussion Symptom Inventory (PCSI-SR8) in children aged 8–12 years

**DOI:** 10.3389/fneur.2024.1372640

**Published:** 2024-03-11

**Authors:** Marina Zeldovich, Leonie Krol, Dagmar Timmermann, Ugne Krenz, Juan Carlos Arango-Lasprilla, Gerard Gioia, Knut Brockmann, Inga K. Koerte, Anna Buchheim, Maike Roediger, Matthias Kieslich, Nicole von Steinbuechel, Katrin Cunitz

**Affiliations:** ^1^Institute of Medical Psychology and Medical Sociology, University Medical Center Goettingen, Goettingen, Germany; ^2^Institute of Psychology, University of Innsbruck, Innsbruck, Austria; ^3^Department of Psychology, Virginia Commonwealth University, Richmond, VA, United States; ^4^Division of Pediatric Neuropsychology, Safe Concussion Outcome Recovery and Education Program, Children's National Health System, Department of Pediatrics and Psychiatry and Behavioral Sciences, George Washington University School of Medicine, Rockville, MA, United States; ^5^Interdisciplinary Pediatric Center for Children with Developmental Disabilities and Severe Chronic Disorders, Department of Pediatrics and Adolescent Medicine, University Medical Center Goettingen, Goettingen, Germany; ^6^Department of Child and Adolescent Psychiatry, Psychosomatics, and Psychotherapy, Ludwig-Maximilians-Universitaet Muenchen, Munich, Germany; ^7^Department of Pediatric and Adolescent Medicine- General Pediatrics- Intensive Care Medicine and Neonatology, University Hospital Muenster, Muenster, Germany; ^8^Department of Paediatric Neurology, Goethe-University Frankfurt/Main, Frankfurt, Germany; ^9^Department of Psychiatry and Psychotherapy, University Medical Center Goettingen, Goettingen, Germany

**Keywords:** pediatric traumatic brain injury, post-concussion symptoms, patient-reported outcome measure (PROM), Postconcussion Symptom Inventory (PCSI), reference values

In the published article, there was an error in Table 5 as published. There was an error in the sixth column containing the test statistic (t) and subsequently in the table footer (t, t-value). The statistic should be z (z-value). The corrected Table 5 and its caption Results of negative binomial regression analyses appear below.

**Table 5:** Results of negative binomial regression analyses.

	**Variable**	**Reference group**	**Estimate**	**S.E**.	***z***	***p***
Total score	(Intercept)	-	2.22	0.26	8.44	**< 0.001**
	Age	-	−0.02	0.02	−0.80	0.421
	Male	Female	−0.12	0.07	−1.66	0.097
	No chronic health complaints	At least one chronic health complaint	−0.57	0.11	−5.40	**< 0.001**
Physical	(Intercept)	-	1.14	0.36	3.14	**0.002**
	Age	-	−0.02	0.03	−0.53	0.596
	Male	Female	−0.13	0.10	−1.33	0.185
	No chronic health complaints	At least one chronic health complaint	−0.58	0.14	−4.04	**< 0.001** ^*^
Emotional	(Intercept)	-	0.91	0.23	3.94	**< 0.001**
	Age	-	−0.03	0.02	−1.15	0.250
	Male	Female	−0.13	0.06	−2.15	**0.032**
	No chronic health complaints	At least one chronic health complaint	−0.39	0.09	−4.46	**< 0.001** ^*^
Cognitive	(Intercept)	-	1.06	0.33	3.16	**0.002**
	Age	-	−0.02	0.03	−0.74	0.461
	Male	Female	−0.09	0.09	−1.02	0.306
	No chronic health complaints	At least one chronic health complaint	−0.78	0.13	−6.13	**< 0.001** ^*^
Fatigue	(Intercept)	-	−0.46	0.42	−1.08	0.281
	Age	-	0.02	0.04	0.41	0.681
	Male	Female	−0.07	0.11	−0.61	0.540
	No chronic health complaints	At least one chronic health complaint	−0.48	0.16	−2.98	**0.003** ^*^

Estimate: regression coefficient; S.E.: standard error; *z*: *z*-value; *p*: *p*-value; values in bold are significant at 5%. Covariates additionally marked with an asterisk (^*^) are significant at 1.25% (adjusted significance level after Bonferroni correction: α_adj_ = 0.05/4 = 0.0125).

In the published article, there was an error in Supplementary Table S2. There was an error in the sixth column containing the test statistic (t) and subsequently in the table footer (t, t-value). The statistic should be z (z-value).

In the published article, there was an error in the Funding statement. The names of two funding organizations were misspelled (i.e., Senckenbergische Stiftung instead of Dr. Senckenbergische Stiftung and Christ'sche Stiftungen instead of Dr. Christ'sche Stiftungen). The correct Funding statement appears below.

