# Supplementary material for: Psychometric evaluation and reference values for the German Postconcussion Symptom Inventory (PCSI-SR8) in children aged 8–12 years
Source: Front Neurol. 2023 Nov 17;14:1266828. doi: 10.3389/fneur.2023.166828 (PMC10693295; doi:10.3389/fneur.2023.166828)
Supplement: Supplementary file 1 [file Data_Sheet_1.pdf]

Supplementary Material

Psychometric Evaluation and Reference Values for the German Postconcussion Symptom Inventory (PCSI-SR8) in Children Aged 8–12 Years

1 Supplementary Tables

Supplementary Table S1. Response patterns of the PCSI-SR8 in the TBI sample (stratified by injury severity) and in the general population sample as well as in the original English language study.

Scale	Item	Response category	TBI sample (N=132)	mild (n = 95)	moderate (n = 11)	severe (n = 26)	General population sample (N = 1047)	Original English version[1] (N = 264)
Physical	Headache	No	80 (60.6%)	59 (62.1%)	7 (63.6%)	14 (53.8%)	694 (34.8%)	32%
		A Little	44 (33.3%)	30 (31.6%)	4 (36.4%)	10 (38.5%)	323 (16.2%)	39%
		A Lot	8 (6.1%)	6 (6.3%)	0 (0%)	2 (7.7%)	30 (1.5%)	28%
	Nausea	No	104 (78.8%)	72 (75.8%)	9 (81.8%)	23 (88.5%)	856 (42.9%)	60%
		A Little	20 (15.2%)	16 (16.8%)	1 (9.1%)	3 (11.5%)	171 (8.6%)	30%
		A Lot	8 (6.1%)	7 (7.4%)	1 (9.1%)	0 (0%)	20 (1.0%)	10%
	Balance problems	No	111 (84.1%)	82 (86.3%)	8 (72.7%)	21 (80.8%)	942 (47.2%)	64%
		A Little	16 (12.1%)	10 (10.5%)	3 (27.3%)	3 (11.5%)	91 (4.6%)	28%
		A Lot	5 (3.8%)	3 (3.2%)	0 (0%)	2 (7.7%)	14 (0.7%)	8%
	Dizziness	No	107 (81.1%)	78 (82.1%)	9 (81.8%)	20 (76.9%)	934 (46.8%)	53%
		A Little	23 (17.4%)	15 (15.8%)	2 (18.2%)	6 (23.1%)	94 (4.7%)	37%
		A Lot	2 (1.5%)	2 (2.1%)	0 (0%)	0 (0%)	19 (1.0%)	10%
	Blurred vision	No	112 (84.8%)	80 (84.2%)	9 (81.8%)	23 (88.5%)	954 (47.8%)	80%
		A Little	17 (12.9%)	12 (12.6%)	2 (18.2%)	3 (11.5%)	82 (4.1%)	18%
		A Lot	3 (2.3%)	3 (3.2%)	0 (0%)	0 (0%)	11 (0.6%)	2%
	Feeling slowed down	No	113 (85.6%)	82 (86.3%)	10 (90.9%)	21 (80.8%)	924 (46.3%)	62%
		A Little	17 (12.9%)	12 (12.6%)	1 (9.1%)	4 (15.4%)	116 (5.8%)	30%
		A Lot	2 (1.5%)	1 (1.1%)	0 (0%)	1 (3.8%)	7 (0.4%)	8%
	Sensitivity to light	No	102 (77.3%)	78 (82.1%)	8 (72.7%)	16 (61.5%)	859 (43.0%)	63%
		A Little	27 (20.5%)	14 (14.7%)	3 (27.3%)	10 (38.5%)	163 (8.2%)	28%
		A Lot	3 (2.3%)	3 (3.2%)	0 (0%)	0 (0%)	25 (1.3%)	10%
	Sensitivity to noise	No	95 (72.0%)	71 (74.7%)	7 (63.6%)	17 (65.4%)	814 (40.8%)	66%
		A Little	31 (23.5%)	20 (21.1%)	4 (36.4%)	7 (26.9%)	194 (9.7%)	24%
		A Lot	6 (4.5%)	4 (4.2%)	0 (0%)	2 (7.7%)	39 (2.0%)	9%
Emotional	Irritability	No	65 (49.2%)	48 (50.5%)	3 (27.3%)	14 (53.8%)	529 (26.5%)	57%
		A Little	61 (46.2%)	43 (45.3%)	7 (63.6%)	11 (42.3%)	462 (23.1%)	33%
		A Lot	6 (4.5%)	4 (4.2%)	1 (9.1%)	1 (3.8%)	56 (2.8%)	10%
	Sadness	No	88 (66.7%)	68 (71.6%)	4 (36.4%)	16 (61.5%)	633 (31.7%)	74%

Scale	Item	Response category	TBI sample (N=132)	mild (n = 95)	moderate (n = 11)	severe (n = 26)	General population sample (N = 1047)	Original English version[1] (N = 264)
		A Little	37 (28.0%)	23 (24.2%)	6 (54.5%)	8 (30.8%)	384 (19.2%)	18%
		A Lot	7 (5.3%)	4 (4.2%)	1 (9.1%)	2 (7.7%)	30 (1.5%)	8%
	**Nervousness**	No	95 (72.0%)	70 (73.7%)	8 (72.7%)	17 (65.4%)	747 (37.4%)	63%
		A Little	30 (22.7%)	20 (21.1%)	2 (18.2%)	8 (30.8%)	272 (13.6%)	31%
		A Lot	7 (5.3%)	5 (5.3%)	1 (9.1%)	1 (3.8%)	28 (1.4%)	6%
**Cognitive**	**Diffculty thinking clearly**	No	100 (75.8%)	75 (78.9%)	8 (72.7%)	17 (65.4%)	808 (40.5%)	57%
		A Little	28 (21.2%)	17 (17.9%)	3 (27.3%)	8 (30.8%)	215 (10.8%)	31%
		A Lot	4 (3.0%)	3 (3.2%)	0 (0%)	1 (3.8%)	24 (1.2%)	12%
	**Difficulty concentrating**	No	86 (65.2%)	66 (69.5%)	5 (45.5%)	15 (57.7%)	640 (32.0%)	52%
		A Little	41 (31.1%)	27 (28.4%)	4 (36.4%)	10 (38.5%)	358 (17.9%)	36%
		A Lot	5 (3.8%)	2 (2.1%)	2 (18.2%)	1 (3.8%)	49 (2.5%)	13%
	**Difficulty remembering**	No	91 (68.9%)	70 (73.7%)	7 (63.6%)	14 (53.8%)	802 (40.2%)	46%
		A Little	37 (28.0%)	25 (26.3%)	3 (27.3%)	9 (34.6%)	217 (10.9%)	43%
		A Lot	4 (3.0%)	0 (0%)	1 (9.1%)	3 (11.5%)	28 (1.4%)	11%
	**Thinking more slowly**	No	108 (81.8%)	80 (84.2%)	9 (81.8%)	19 (73.1%)	873 (43.7%)	56%
		A Little	18 (13.6%)	12 (12.6%)	1 (9.1%)	5 (19.2%)	154 (7.7%)	32%
		A Lot	6 (4.5%)	3 (3.2%)	1 (9.1%)	2 (7.7%)	20 (1.0%)	11%
**Fatigue**	**Fatigue**	No	100 (75.8%)	73 (76.8%)	7 (63.6%)	20 (76.9%)	754 (37.8%)	34%
		A Little	25 (18.9%)	16 (16.8%)	3 (27.3%)	6 (23.1%)	262 (13.1%)	42%
		A Lot	7 (5.3%)	6 (6.3%)	1 (9.1%)	0 (0%)	31 (1.6%)	23%
	**Drowsiness**	No	107 (81.1%)	76 (80.0%)	10 (90.9%)	21 (80.8%)	886 (44.4%)	43%
		A Little	19 (14.4%)	14 (14.7%)	0 (0%)	5 (19.2%)	143 (7.2%)	40%
		A Lot	6 (4.5%)	5 (5.3%)	1 (9.1%)	0 (0%)	18 (0.9%)	17%

[1] Values obtained from the original validation study by Sady et al. (2014). According to Sady et al. (2014), values do not always add up to 100% due to rounding. Therefore, no additional absolute frequencies were calculated.

Note. N = sample frequencies, n = subgroup frequencies.

Supplementary Table S2. Results of negative binomial regressions with second order interactions.

	Variable	Reference Group	Estimate	S.E.	*z*	*p*
Total score	(Intercept)	-	1.46	0.74	1.97	0.049
	Age	-	0.05	0.08	0.69	0.492
	Male	Female	0.11	0.53	0.21	0.836
	No chronic health complaints	At least one chronic health complaint	0.23	0.74	0.31	0.759
	Age*Male	Age*Female	-0.01	0.05	-0.23	0.819
	Age*No chronic health complaints	Age*At least one chronic health complaint	-0.07	0.08	-0.99	0.323
	Male*No chronic health complaints	Female*At least one chronic health complaint	-0.13	0.21	-0.60	0.549
Physical	(Intercept)	-	1.11	1.00	1.10	0.271
	Age	-	-0.03	0.10	-0.25	0.799
	Male	Female	-0.40	0.73	-0.55	0.580
	No chronic health complaints	At least one chronic health complaint	-0.28	1.01	-0.28	0.779
	Age*Male	Age*Female	0.05	0.07	0.71	0.480
	Age*No chronic health complaints	Age*At least one chronic health complaint	-0.02	0.10	-0.17	0.865
	Male*No chronic health complaints	Female*At least one chronic health complaint	-0.25	0.29	-0.85	0.396
Emotional	(Intercept)	-	-0.43	0.61	-0.70	0.484
	Age	-	0.11	0.06	1.71	0.088
	Male	Female	0.11	0.47	0.23	0.815
	No chronic health complaints	At least one chronic health complaint	1.09	0.62	1.77	0.077
	Age*Male	Age*Female	-0.02	0.04	-0.36	0.718
	Age*No chronic health complaints	Age*At least one chronic health complaint	-0.14	0.06	-2.34	**0.019**
	Male*No chronic health complaints	Female*At least one chronic health complaint	-0.10	0.18	-0.60	0.552
Cognitive	(Intercept)	-	-0.03	0.88	-0.04	0.968
	Age	-	0.08	0.09	0.92	0.356
	Male	Female	0.56	0.67	0.84	0.402
	No chronic health complaints	At least one chronic health complaint	0.16	0.89	0.18	0.857
	Age*Male	Age*Female	-0.06	0.06	-0.91	0.365
	Age*No chronic health complaints	Age*At least one chronic health complaint	-0.09	0.09	-1.01	0.313
	Male*No chronic health complaints	Female*At least one chronic health complaint	-0.09	0.26	-0.36	0.722
Fatigue	(Intercept)	-	-1.03	1.11	-0.93	0.355
	Age	-	0.08	0.11	0.71	0.478

Variable	Reference Group	Estimate	S.E.	*z*	*p*
Male	Female	0.85	0.84	1.01	0.314
No chronic health complaints	At least one chronic health complaint	-0.40	1.13	-0.36	0.720
Age*Male	Age*Female	-0.10	0.08	-1.29	0.197
Age*No chronic health complaints	Age*At least one chronic health complaint	-0.01	0.11	-0.13	0.898
Male*No chronic health complaints	Female*At least one chronic health complaint	0.14	0.32	0.44	0.659

Note. *: interaction between the variables; Estimate: regression coefficient; S.E.: standard error; *z: z*-value; *p*: *p*-value; values in **bold** are significant at 5%, but not at 1.25% (adjusted significance level after Bonferroni correction: α_{adj} = 0.05/4 = 0.0125).
